# Idiopathic environmental intolerance attributed to electromagnetic fields (IEI-EMF): A systematic review of identifying criteria

**DOI:** 10.1186/1471-2458-12-643

**Published:** 2012-08-11

**Authors:** Christos Baliatsas, Irene Van Kamp, Erik Lebret, G James Rubin

**Affiliations:** 1 Institute for Risk Assessment Sciences, Utrecht University, Utrecht, The Netherlands; 2 National Institute for Public Health and the Environment (RIVM), Bilthoven, The Netherlands; 3 King’s College London, Department of Psychological Medicine, Institute of Psychiatry, London, UK

## Abstract

**Background:**

Idiopathic environmental intolerance attributed to electromagnetic fields (IEI-EMF) remains a complex and unclear phenomenon, often characterized by the report of various, non-specific physical symptoms (NSPS) when an EMF source is present or perceived by the individual. The lack of validated criteria for defining and assessing IEI-EMF affects the quality of the relevant research, hindering not only the comparison or integration of study findings, but also the identification and management of patients by health care providers. The objective of this review was to evaluate and summarize the criteria that previous studies employed to identify IEI-EMF participants.

**Methods:**

An extensive literature search was performed for studies published up to June 2011. We searched EMBASE, Medline, Psychinfo, Scopus and Web of Science. Additionally, citation analyses were performed for key papers, reference sections of relevant papers were searched, conference proceedings were examined and a literature database held by the Mobile Phones Research Unit of King’s College London was reviewed.

**Results:**

Sixty-three studies were included. “Hypersensitivity to EMF” was the most frequently used descriptive term. Despite heterogeneity, the criteria predominantly used to identify IEI-EMF individuals were: 1. Self-report of being (hyper)sensitive to EMF. 2. Attribution of NSPS to at least one EMF source. 3. Absence of medical or psychiatric/psychological disorder capable of accounting for these symptoms 4. Symptoms should occur soon (up to 24 hours) after the individual perceives an exposure source or exposed area. (Hyper)sensitivity to EMF was either generalized (attribution to various EMF sources) or source-specific. Experimental studies used a larger number of criteria than those of observational design and performed more frequently a medical examination or interview as prerequisite for inclusion.

**Conclusions:**

Considerable heterogeneity exists in the criteria used by the researchers to identify IEI-EMF, due to explicit differences in their conceptual frameworks. Further work is required to produce consensus criteria not only for research purposes but also for use in clinical practice. This could be achieved by the development of an international protocol enabling a clearly defined case definition for IEI-EMF and a validated screening tool, with active involvement of medical practitioners.

## Background

Although the issue of idiopathic intolerances attributed to environmental exposures (IEI) first appeared in the scientific literature more than five decades ago [1], the possible underlying causes, as the term “idiopathic” suggests, remain unclear [2] and there is no widely accepted protocol for the identification of patients and treatment [3]. A representative example is the variety of physical symptoms without a clear pathological basis that are attributed by the patients to relatively low-level exposure to non-ionizing electromagnetic fields (EMF), emitted by sources such as mobile phone devices and base stations, high-voltage overhead powerlines, computer equipment and domestic appliances [4]. This phenomenon is better known within the public and scientific context as "electromagnetic hypersensitivity"(EHS), although since 2005 the term “Idiopathic Environmental Intolerance Attributed to EMF" (IEI-EMF) has been proposed by the World Health Organization (WHO) as an etiologically neutral description [5]. In this paper, the descriptive term “IEI-EMF” is used.

According to the WHO [5], people with IEI-EMF are mainly characterized by the report of non-specific physical symptoms (NSPS), without a consistent pattern [6], such as redness, tingling, burning sensations in the facial area, fatigue, tiredness, lack of concentration, dizziness, nausea, heart palpitation and digestive disturbances. IEI-EMF is often accompanied by occupational, social and mental impairment [4,7] and its estimated prevalence varies considerably, probably due to different methodological approaches; 1.5% in Sweden [6], 3.2% in California [8], 3.5% in Austria [9], 5% in Switzerland [10] and 13.4% in Taiwan [11]. Demographic characteristics such as age, gender and occupational status have repeatedly been associated with IEI-EMF [6,10].

The experience and belief of IEI-EMF patients is in contrast with the scientific state of the art; results from systematic assessment of experimental and epidemiological evidence are consistent, concluding that a causal association of EMF exposure with symptomatic and other physiologic or cognitive reactions cannot be adequately supported [12-17]. IEI-EMF has been associated with psychological components [18-23] but their exact role is not clear. Although a possible effect of exposure cannot yet be ruled out because of methodological obstacles in research primarily regarding exposure assessment and study design [14,16], more recent approaches stress the importance of looking into the interaction of environmental, biological, psychological and social pathways [24].

However, it is still controversial who should be categorised as having IEI-EMF. The lack of a validated, mutually accepted case definition and diagnostic instrument affects the quality of the research outcomes and increases the methodological heterogeneity, resulting in limited comparability between the studies. That stands in the way of a reliable estimation of the prevalence of IEI-EMF in the general population, proper meta-analysis of etiological evidence, the identification of health outcome patterns/profiles and contributes to a great deal of uncertainty regarding the characteristics, identification and management of this sensitivity by health care providers [25-27].

No systematic review has been performed yet focusing on the existing definitions and criteria for the identification of people with IEI-EMF. In light of the need to inform health care profesionals about relevant aspects of IEI-EMF and prepare the ground for discussion and consensus in the research community on widely supported case definition criteria, the present paper identified the relevant studies on IEI-EMF published to date, in order to summarize:

• The descriptive terms used to define IEI-EMF.

• The inclusion criteria and procedure for the identification of individuals with IEI-EMF.

## Methods

### Search strategy for the identification of studies

Initially, the following electronic databases were searched to detect relevant studies that were published from inception to April 2010: Embase (Elsevier B.V., Amsterdam, The Netherlands), Medline (US National Library of Medicine, Bethesda, Maryland), PsychInfo (American Psychological Association, Washington, DC). Web of Knowledge (Institute for Scientific Information, The Thomson Corporation, Stamford, Connecticut) and Scopus (Elsevier B.V., Amsterdam, The Netherlands). A wide range of (combined) keywords was used with regards to EMF exposure, sensitivity and related health outcomes, which is presented in Table 1. In addition to the electronic database searches, the reference sections of previous systematic reviews, key papers, international reports on EMF and health and research databases of websites focused on the issue of EMF such as the “EMF Portal” and the WHO webpage were checked for potentially relevant articles. A wide literature database held by the Mobile Phones Research Unit of King’s College London was also consulted. A second literature search was carried out in order to update our review with studies published from May 2010 to June 2011.

**Table 1:** Key search terms

	
**Sensitivity:**	Electrosensitivity, Electromagnetic hypersensitivity, Electrical sensitivity, Electromagnetic sensitivity, Electric hypersensitivity, IEI-EMF, Environmental intolerance, environmental illness.
**Exposure:**	EMF, ELF, Electromagnetic field(s), Electromagnetic exposure, mobile telephones, mobile phone(s), Base stations, Powerlines, Celltowers, Antenna(e), UMTS, GSM, DECT, VDU, cell phones.
**Health Outcome:**	Symptom(s), well-being, attributed symptoms, headache, fatigue.
**Time period**	From inception – 2011.

### Inclusion criteria

Only primary studies written in English and published in the peer-reviewed literature were considered as suitable for inclusion in the current review. Conference presentations, brief communications and reviews were excluded. The primary condition to include a study was the report of use of at least one criterion to identify individuals with IEI-EMF. Studies focusing on health effects from wider environmental exposures (such as chemicals) were eligible as long as they attempted to identify sensitivity to EMF in their investigation. Studies recruiting exclusively “healthy” individuals without any attempt to assess IEI-EMF or identify relevant individuals were excluded. Since the “attribution” of health complaints to EMF is not necessarily synonymous with IEI-EMF and it is not an established prerequisite for its existence, studies relying solely on “attribution” without any mention of and explicit conceptual link with IEI-EMF or synonymous terms were not considered eligible for this review. Among papers based on the same sample and identifying criteria of IEI-EMF, the first publication was included.

### Data extraction

For each included study, the following data were abstracted: reference and country, study design, methods and source of sample recruitment, IEI-EMF sample characteristics (such as sample size, age mean or range and gender distribution), type of sensitivity based on the triggering EMF source(s), the criteria used to identify individuals with IEI-EMF, exclusion criteria (based on self-report/interview or clinical examination) and the case definition procedure followed for the identification of IEI-EMF (such as self-report and/or medical examination to exclude the possibility that a diagnosed disorder was responsible for the reported health complaints) (Tables 2 and 3). The data provided in the tables were derived from the information that was given or could be inferred from the original publications. However, in some cases (part of) the necessary information was not provided in the reviewed articles.

**Table 2:** Experimental studies on IEI-EMF

**Reference**	**Study design**	**Recruitment**	**Type of sensitivity**	**IEI-EMF sample characteristics**	**Identifying criteria for IEI-EMF**	**Main exclusion criteria**	**Identification/Case definition methods for IEI-EMF**
Rea et al., 1991 (USA) [28].	Provocation	Voluntary participation.	General	N = 100.	Self-reported sensitivity to EMF.	N.R/E.	Subjective report, medical examination.
Hamnerius et al., 1993(Sweden) [29].	Provocation	IEI-EMF subjects referred to a health care service/institution.	VDU-specific	N = 30.	Report of “distinctive” symptoms, occurrence of the symptoms within an hour from being exposed to VDU and disappearance or substantial reduction within a few hours after exposure termination.	Somatic or psychiatric disorder that could account for the reported symptomatology.	Subjective report, medical, psychiatric & psychological examination.
Arnetz et al., 1995 (Sweden) [30].	Intervention	IEI-EMF subjects referred to a health care service/institution.	General	N = 20, m.a = 45, f.g = 75%.	Report of symptoms attributed to EMF exposure.	Somatic or psychiatric disorder that could account for the reported symptomatology.	Subjective report, medical & psychiatric examination.
Andersson et al., 1996 (Sweden) [31].	Provocation	IEI-EMF subjects referred to a health care service/institution.	General	N = 17, m.a = 41.7, f.g = 70.6%.	Experience of “typical” symptoms of “electric hypersensitivity” when being in an “electrical environment”, duration of at least 6 months, limitations in daily functioning, experience of symptomatology within less than 30 minutes after exposure to electric equipment under experimental testing.	Somatic or psychiatric disorder that could account for the reported symptomatology.	Subjective report, medical & psychiatric examination.
Bertoft et al., 1996 (Sweden) [32].	Provocation	Voluntary participation of IEI-EMF subjects.	General	N = 5, a.r = 46-60, f.g = 80%.	Self-reported hypersensitivity to EMF.	N.R/E.	Subjective report.
Toomingas, 1996 (Sweden) [33].	Provocation (case study)	Voluntary participation of a medical patient.	General	N = 1 male, age = 35.	Fear of a negative impact of health caused by EMF exposure, report of symptoms attributed to EMF such as fatigue, headache, lack of concentration, numbness & paresthesia in the arms, greasy feeling in the palms, inability to work due to the reported symptoms.	N.R/E.	Subjective report, medical & neurological examination.
Sandström et al., 1997 (Sweden) [34].	Provocation	IEI-EMF subjects treated to a health care service/institution.	VDT & fluorescent light-specific	N = 10, m.a = 47, f.g = 70%.	Self-report of a combination of skin (mucous), eye & neurological symptoms, attribution of these symptoms to EMF emitted from VDT work, fluorescent light or TV.	Somatic or psychiatric diseases severe enough to require medical treatment.	Subjective report, medical examination.
Hillert et al., 1998 (Sweden) [35].	Intervention	IEI-EMF subjects referred to a health care service/institution.	General	N = 10, m.a = 40, f.g = 60%.	Self-reported hypersensitivity to EMF, age between 18–65 y.o, being employed for at least 1 week during the past 3 months, symptoms had to show some variation due to perceived exposure to EMF or proximity to relative equipment.	Medical or mental disorder that could account for the reported symptomatology, long period of sick leave, unemployment.	Subjective report, medical examination.
Trimmel et al., 1998 (Austria) [36].	Provocation	Voluntary participation.	General	N = 36, a.r = 18-36.	Individual belief of an “exceptional reaction to EMF”, a score of >50 on a continuous rating scale between 0–100.	N.R/E.	Subjective report.
Flodin et al., 2000 (Sweden) [37].	Provocation	IEI-EMF subjects who were members of a relative self-group or registered to a health care service/institution.	General	N = 15, m.a = 48.3, f.g = 73.3.	Attribution of symptoms to named EMF sources, mean reaction should occur within 60 minutes of exposure, there was no experience of symptoms at home or workplace when the subject was considered as “unexposed”, symptoms should disappear within a few days after exposure.	Slow reaction or denial for participation because of symptom severity, undergoing treatment for medical conditions.	Subjective report, medical examination (registered IEI-EMF subjects).
Lohne-Rahm et al., 2000 (Sweden) [38].	Provocation	Voluntary participation after description of the study in newspaper advertisements or IEI-EMF subjects referred to a health care service/institution.	General	N = 12.	Report of skin symptoms during a 30-minute exposure to EMF & symptom duration of at least 6 months.	Diagnosed skin diseases, slow, excessive or no reactions during the experiment.	Subjective report, medical examination (referred IEI-EMF subjects).
Hillert et al., 2001 (Sweden) [39].	Intervention	IEI-EMF subjects referred to a health care service/institution.	General	N = 16, m.a = 39.5, f.g = 81.3%.	Self-reported hypersensitivity to electricity, experience of change in symptoms within 24 hours after a perceived change in exposure to EMF, a history of VDU or fluorescent lights as the initial triggering factors.	Somatic or psychological disorder that could account for the reported symptomatology.	Subjective report, medical & psychological examination.
Lyskov et al., 2001 (Sweden) [40].	Provocation	IEI-EMF subjects referred to a health care service/institution.	General	N = 20, m.a = 45.8, f.g = 75%.	Self-reported hypersensitivity to EMF	N.R/E.	Subjective report, medical examination.
Hietanen et al., 2002 (Finland) [41].	Provocation	Voluntary participation.	General	N = 20, m.a = 49, f.g = 65%.	Self-reported hypersensitivity to EMF, experience of symptoms during a 30-minute (provocation) test period.	N.R/E.	Subjective report, medical examination.
Hillert et al., 2002 (Sweden) [42].	Intervention	IEI-EMF subjects referred to a health care service/institution.	General	N = 22, m.a = 42, f.g = 64%.	Report of symptoms assumed to be caused by sensitivity to EMF.	Medical or psychological condition that could account for the symptoms.	Subjective report, medical & psychiatric examination.
Mueller et al., 2002 (Switzerland) [43].	Provocation	Voluntary participation.	General	N = 63, m.a = 49.5, f.g = 51%.	Self-reported sensitivity to EMF or “Electrical Hypersensitivity Syndrome (EHS)”.	N.R/E.	Subjective report.
Leitgeb et al., 2003(Austria) [44].	Provocation	Randomly selected sample from general population (N = 708).	General	a.r = 17-60.	Increased levels of “electrosensibility”, defined as the individual ability to perceive electric or electromagnetic exposures without necessarily developing health symptoms.	N.R/E.	Measurement of EMF perception thresholds.
Österberg et al., 2004 (Sweden) [45].	Provocation	Randomly selected sample from general population (N = 13381), based on Östergren et al. (report) [46].	General	N = 16, m.a = 41.8, f.g = 50%.	Individual experience the past 2 weeks of “very much” physiologic “annoyance” attributed to FTL, and/or VDU and/or other electrical equipment.	Report of long-term sick leave, disability pension, subjects diagnosed with severe medical condition that required medication (e.g. diabetes), age of >58 y.o	Subjective report, medical examination.
Belyaev et al., 2005 (Sweden) [47].	Provocation	Voluntary participation.	General	N = 7, m.a = 44.8, f.g = 71.5%.	Self-reported hypersensitivity to EMF.	Smoking, regular medication	Subjective report.
Frick et al., 2005 (Germany) [48].	Provocation	Voluntary participation after description of the study in a local newspaper.	General	N = 30, m.a = 41.7, f.g = 77%.	Self-reported hypersensitivity to named EMF sources, attribution of severe symptoms that limited daily functioning & age between 18–64 y.o.	Not complaining or not experiencing limitations to daily living due to the reported symptomatology.	Subjective report.
Wenzel et al., 2005 (Germany) [49].	Provocation	Voluntary participation.	VDU & powerline-specific	N = 3 male subjects, m.a = 37.	Concern about the effects of EMF exposure, report of various symptoms attributed to VDU and/or powerlines, abstinence from smoking.	N.R/E.	Subjective report.
Regel et al., 2006 (Switzerland) [50].	Provocation	Voluntary participation after description of the study in advertisements in a local newspaper, flyers & use of databases of two previous studies with IEI-EMF subjects willing to participate in future research projects.	Base station-specific	N = 33, m.a = 37.7, f.g = 57.5%.	Self-reported sensitivity to EMF emitted by mobile or cordless phones & antennas.	Regular consumption of narcotics or psychoactive drugs in the last 6 months, smoking, diagnosed with a chronic disease, pregnancy, medical history of head injuries, neurologic/psychiatric diseases, sleep disturbances, average alcohol consumption of >10 drinks per week, average consumption of caffeinated beverages amounting to >450 milligrams caffeine per day, shift workers, undertaking long-haul flights of >3 hours time zone difference within the last month.	Subjective report.
Rubin et al., 2006 (UK) [51].	Provocation	Through mailshots organised by an IEI-EMF support group, advertisements & articles in health care institutions & practices.	MP-specific	N = 71, m.a = 37.1, f.g = 56%.	Frequent experience of headache-related symptoms within 20 minutes of using a 900 MHz GSM MP.	Age of <18 or >75 y.o, pregnancy, psychotic illness, use of antidepressants, report of severe symptoms at baseline while in the testing room.	Subjective report.
Eltiti et al., 2007 (UK) [52].	Provocation	Voluntary participation through local advertising, IEI-EMF action groups & word of mouth.	MP & base station-specific	N = 56, m.a = 46.1, f.g = 42.9%.	Individual experience of negative health effects attributed to EMF emitted from mobile phone devices and/or base stations, based on the “Electromagnetic Hypersensitivity Questionnaire” [53].	History of brain injury, currently suffering from epilepsy or claustrophobia, undergone treatment for mental disease or psycho-active medication within 4 months before the study.	Subjective report.
Schröttner et al., 2007 (Austria) [54].	Provocation	Three different recruitment sources: 1. EMF self-help groups. 2. Through advertisements in local newspapers & inviting patients that contacted a health care service/institution for their EMF-attributed symptoms. 3. Subjects reporting severe sleep problems being deeply convinced that these were caused by EMF exposure.	General	Recruitment 1: N = 37, a.r = 27-81, f.g = 67.6%. Recruitment 2: N = 29, a.r = 32-63, f.g = 79%. Recruitment 3: N = 24, a.r = 37-73, f.g = 62.5%.	Self-reported hypersensitivity to electricity, attribution of symptoms to EMF, active avoidance behavior to EMF sources.	Sensitivity only to sources of flickering light such as VDU fluorescent tubes.	Medical examination (for part of the group of “Recruitment 2”).
Bamiou et al., 2008 (UK) [55].	Provocation	Voluntary participation after description of the study through advertisements at a health care services/institutions & relative website & short film shown on the national television.	MP-specific	N = 9, m.a = 36.7, f.g = 66.7.	Report of headache and/or disorientation, dizziness, muzziness, nausea attributed to mobile telephone use, age between 20–55 y.o, normal tympanometry & normal pure tone audiometric thresholds in both ears.	N.R/E.	Subjective report, audiometric examination.
Hillert et al., 2008 (Sweden) [56].	Provocation	Voluntary participation after description of the study in newspapers, or individual initiative.	MP-specific	N = 38, m.a = 28, f.g = 63.2%.	Report of headache, vertigo or other kind of pain or discomfort in the head attributed to MP use.	Attribution of symptoms to sources other than MP, medical or psychological illness, undergoing medication, sleep disorders, hypertension, pregnancy, history of severe injury.	Subjective report.
Kwon et al., 2008 (Finland) [57].	Provocation	Voluntary participation after description of the study in an advertisement that announced a monetary prize.	MP-specific	N = 2 male subjects, m.a = 37.	Report of suffering from severe symptomatology after use of a mobile phone, high score on a scale on EMF sensibility (defined as the individual ability to perceive EMF without necessarily developing symptoms).	Neurological disease, auditory abnormality, being on permanent medication.	Subjective report.
Landgrebe et al., 2008a (Austria & Germany) [58].	Provocation	Voluntary participation after description of the study in newspapers and informative events at public locations and institutions.	General	N = 88, m.a = 50.5, f.g = 58.4%.	A symptom score of at least 19 points on the “Regensburger EMF complaint list” [59], attribution of health symptoms to named EMF sources & age between 18–75 y.o	Unstable medical condition.	Subjective report.
Leitgeb et al., 2008 (Austria & Germany) [60].	Crossover field study	Voluntary participation after description of the study in media.	General	N = 43, m.a = 55.5, f.g = 60.5%.	Personal conviction on a causal role of EMF indicated by the employment of precautionary activities and/or measures (e.g. reducing fields, measuring exposure in the household etc.), above-normal symptom scores on standardized questionnaires such as the “Freiburger Personality Inventory” [61] and “PSQI” [62] (at least 5 points on the latter).	Neurological & psychiatric disorders, somatic conditions that could account for sleep disturbances, drug consumption less than 2 weeks before the study, medical treatment for severe conditions.	Subjective report.
Augner et al., 2009 (Austria) [63].	Provocation plus cross-sectional data	Voluntary participation.	General	N = 8, a.r = 18-67.	Self-reported electromagnetic hypersensitivity (rated as “strong” or “very strong”).	N.R/E.	Subjective report.
Furubayashi et al., 2009 (Japan) [64].	Provocation	Randomly selected female subjects (N = 2472).	MP & base station-specific	N = 11, m.a = 37.3.	Report of symptoms attributed to MP use and/or exposure to base stations, symptoms should persist “always” or “almost always”	History of myocardial infarction epilepsy or other (psycho) pathological condition, undergoing medical treatment for severe medical conditions.	Subjective report.
Nam et al., 2009 (South Korea) [65].	Provocation	Voluntary participation after description of the study through advertisements at a health care service/institution.	MP-specific.	N = 18, m.a = 26.1, f.g = 55.5%.	Self-reported hypersensitivity to EMF emitted only by CDMA cellular phones.	Self-reported hypersensitivity to other EMF sources, subjects concerned with payment for volunteering,	Subjective report.
Szemerszky et al., 2010 (Hungary) [66].	Provocation	Voluntary participation of university students.	General	N.R.	Self-reported electrosensitivity (rated from “not at all” to “fully”).	Severe medical disorders, health conditions such as premenstrual syndrome and common cold that could account for the reported symptomatology	Subjective report.
Nieto-Hernandez et al., 2011 (UK) [67].	Provocation	Voluntary participation after description of the study within UK Police Forces with the use of circular emails, notices in police newsletters and intranet sites & advertisements in police-related magazines & websites.	TETRA-specific	N = 60, m.a = 35.6, f.g = 11.7%.	Report of symptoms attributed to TETRA, report of being at least 70 % sure that the radio signal was the responsible source, occurrence of symptoms/sensations within an hour of radio use and when the radio was used near the head.	Pregnancy/trying to conceive, medical or psychological condition which could account for similar symptoms.	Subjective report.

*Although the studies of Österberg et al. [45] and Carlsson et al. [7] are based on the same sample [46], they have some differences in terms of inclusion criteria and/or identification methods.

Abbreviations: N.R., Not reported; N.R/E, Not reported or employed; EMF, Electromagnetic fields; IEI-EMF, Idiopathic environmental intolerance attributed to EMF; m.a, Mean age; a.r, Age range; f.g, Female gender distribution; y.o; Years old; MP, Mobile phone(s); VDT, Video display terminal: VDU, Video display units; GSM, Global system for mobile communications; CDMA, Code division multiple access; TETRA, Terrestrial trunked radio; PSQI, Pittsburgh sleep quality index.

**Table 3:** Observational studies on IEI-EMF

**Reference (Country)**	**Study design**	**Recruitment**	**Type of sensitivity**	**IEI-EMF sample characteristics**	**Identifying criteria for IEI-EMF**	**Main exclusion criteria**	**Identification/Case definition methods for IEI-EMF**
Bergdahl et al., 1998 (Sweden) [68].	Cross-sectional	IEI-EMF subjects referred to a health care service/institution.	General, VDU-specific	N = 28, m.a = 45.5, f.g = 50%.	Report of symptoms assumed to be caused by VDU and/or other EMF sources.	N.R/E.	Subjective report, medical examination.
Hocking, 1998 (Australia) [69].	Cross-sectional	Voluntary participation after description of the study in a medical journal.	General	N = 0 (people identified with IEI-EMF)	Self-reported electrosensitivity.	N.R/E.	Subjective report
Hillert et al., 1999 (Sweden) [70].	Case–control	Subjects selected from an older occupational health survey & IEI-EMF subjects referred to a health care service/institution.	General	N = 62, a.r = 20 ≤ .	Self-reported hypersensitivity to EMF.	N.R/E.	Subjective report, medical examination (referred IEI-EMF subjects).
Stockenius et al., 2000 (Switzerland) [71].	Cross-sectional	Voluntary participation of male subjects (mostly university students) after description of the study through advertisements.	General	N.R.	Self-reported electrosensitivity to named sources (ranked from “very insensitive” to “very sensitive”).	N.R/E.	Subjective report.
Bergdahl et al., 2001 (Sweden) [72].	Cross-sectional	IEI-EMF subjects referred to a health care service/institution.	General	N = 44, m.a = 47, f.g = 57%.	Report of symptoms assumed to be caused by “abnormal sensitivity to EMF”.	N.R/E.	Subjective report, medical interview & examination.
Hillert et al., 2001 (Sweden) [73].	Cross-sectional	IEI-EMF subjects referred to a health care service/institution.	General	N = 14, m.a = 46, f.g = 64.3%.	Self-reported hypersensitivity to EMF including disabling fatigue attributed to EMF exposure.	Medical condition that could account for the reported symptomatology.	Subjective report, medical examination.
Lyskov et al., 2001 (Sweden) [74].	Case–control	IEI-EMF subjects referred to a health care service/institution.	General	N = 20, m.a = 47, f.g = 55%.	Report of a combined pattern of skin, general and ocular symptoms & attribution to EMF exposure.	Chronic diseases, acute illness the last 6 months, undergoing hormonal, hypotensive or sedative therapy.	Subjective report, medical examination.
Hillert et al., 2002 (Sweden) [6].	Cross-sectional	Randomly selected sample from general population (N = 10605).	General	N = 167, a.r = 19-80, f.g = 62.8%	Self-reported hypersensitivity to named EMF sources.	N.R/E.	Subjective report.
Levallois et al., 2002 (USA) [8].	Cross-sectional	Randomly selected sample from general population (N = 2072).	General	N = 68, m.a = 43.4, f.g = 58.8%.	Report of being allergic or very sensitive when being near electrical devices, computers and/or powerlines.	N.R/E.	Subjective report
Stenberg et al., 2002 (Sweden) [75].	Cross-sectional	IEI-EMF subjects referred to a health care service/institution.	General, VDT-specific.	General sensitivity: N = 50, m.a = 49, f.g = 62%. VDT specific: N = 200, m.a = 50, f.g = 78.5%.	General: Experience of symptoms attributed to EMF sources in general within 24 hours after being exposed. VDT-specific: Experience of (mainly skin) symptoms attributed to VDT, TV screens & fluorescent light within 24 hours after being exposed. For all subjects, the possible association between exposure & symptoms could not be ruled out.	Lack of medical records or examination, diagnosed medical condition, no symptom attribution to EMF within 24 hours after being exposed.	Subjective report, medical records & examination.
Sandström et al., 2003 (Sweden) [76].	Case control	IEI-EMF subjects registered to a health care service/institution.	General	N = 14, m.a = 48.9, f.g = 64.3%.	Individual perception that exposure to VDT, FTL, TV and/or other EMF sources causes symptoms within 24 h, the possible exposure-outcome association could not be ruled out.	Symptoms indicating autonomic nervous dysregulation, undergoing hormonal or hypotensive therapy, having arrhythmia due to frequent non-sinus beats or severe cardiac conduction disturbances.	Subjective report, medical examination.
Bergdahl et al., 2004 (Sweden) [77].	Case–control	IEI-EMF subjects referred and registered to a health care service/institution.	General	N = 250, m.a = 49.1, f.g = 75.2%.	Individual perception that exposure to VDT, TV and/or other EMF sources causes symptoms within 24 h.	N.R/E.	Subjective report, medical examination.
Röösli et al., 2004 (Switzerland) [4].	Cross-sectional	The survey was described to various local institutions and organizations which informed & encouraged IEI-EMF subjects to participate.	General	N = 394, m.a = 51, f.g = 57%.	Report of symptoms (open question) attributed to EMF exposure.	N.R/E.	Subjective report.
Bergdahl et al., 2005 (Sweden) [78].	Case–control	IEI-EMF subjects referred to a health care service/institution.	General	N = 33, m.a = 47, f.g = 51.5%.	Report of symptoms assumed to be caused by sensitivity to EMF.	N.R/E.	Subjective report, psychological examination.
* Carlsson et al., 2005 (Sweden) [7].	Cross-sectional	Randomly selected sample from general population (N = 13381), based on Östergren et al. (report) [46].	General	N = 2748 (“some annoyance” by EMF), N = 354 (“much annoyance” by EMF), a.r = 18 ≤ .	Individual experience the past 2 weeks of “some” or “much” physiologic “annoyance” attributed to FTL, and/or VDU and/or other electrical equipment.	N.R/E.	Subjective report.
Eriksson et al., 2006 (Sweden) [79].	Cross-sectional	Random sample from general population (N = 2154).	General	N = 46, a.r = 18-64, f.g =76%.	For the past 3 months, report of 5 symptoms on a weekly basis and 5 on a monthly basis: These symptoms could be: fatigue, feeling heavy-headed, headache, concentration difficulties, itching, burning or irritation of the eyes, dry eyes, dry facial skin, flushed facial skin, itching/stinging/tight or burning sensation in facial skin & cold hands or feet.	N.R/E.	Subjective report.
Schreier et al., 2006 (Switzerland) [10].	Cross-sectional	Randomly selected sample from general population (N = 2048).	General	N = 107, a.r = 14<, f.g = 54.5%.	Report of adverse health effects (open question) attributed to EMF at the time of the interview or anytime in the past.	N.R/E.	Subjective report.
Schüz et al., 2006 (Germany) [80].	Cross-sectional	Voluntary participation from EMF self-help & action groups, internet & media advertisements, invitation letters.	General	N = 107, f.g = 54%.	Self-reported hypersensitivity to EMF.	N.R/E.	Subjective report.
Eltiti et al., 2007 (UK) [53].	Three cross-sectional investigations.	Investigation 1&3: IEI-EMF subjects through local self-help & action groups or personal contact. Investigation 2: Random selection from the general population (N = 3633) .	General	Investigation 1: N = 50, m.a = 52.5, f.g = 66%. Investigation 2: N = 698. Investigation 3: N = 88, m.a = 48.7, f.g = 53.4%.	Investigation 1: Self-reported sensitivity to EMF, attribution of symptoms to EMF. Investigation 2 &3: Self-reported sensitivity to EMF.	N.R/E.	Subjective report.
Landgrebe et al., 2007 (Germany) [81].	Case–control	Voluntary participation after description of the study in a local newspaper.	General	N = 23, m.a = 41.3, f.g = 74%.	Report of severe symptoms that limited daily functioning, attribution of these symptoms to named EMF sources & age between 18–64 y.o.	N.R/E.	Subjective report.
Hardell et al., 2008 (Sweden) [82].	Case–control	Voluntary participation.	General	N = 13 female subjects, m.a = 53.	Report of symptoms attributed to EMF.	Severe medical condition.	Subjective report, medical examination.
Lidmark et al., 2008 (Sweden) [83].	Cross-sectional, plus qualitative data	Voluntary participation of members of an IEI-EMF self-help group.	General	N.R.	Report of symptoms attributed to EMF	N.R/E.	Subjective report, medical & psychiatric examination.
Schröttner et al., 2008 (Austria) [9].	Cross-sectional	Randomly selected sample from general population (N = 526).	General	N = 16, a.r = 15-80, f.g = 50%.	Report of disturbance/adverse health effects (open question) attributed to named EMF sources, looking for medical help because of symptom severity.	N.R/E.	Subjective report.
Dahmen et al., 2009 (Germany) [84].	Case–control	Sample selected from EMF self-help groups, an internet-based survey on EMF and health & local advertisements.	General	N = 132, m.a = 51.5, f.g = 68.2%.	A symptom score of at least 14 points on the “Regensburger EMF complaint list” [85], attribution of health symptoms to named EMF sources & age between 18–56 y.o	Acute psychiatric disorder (after psychiatric examination).	Subjective report.
Johansson et al., 2010 (Sweden) [23].	Case–control	Voluntary participation after description of the study in newspaper advertisements.	General, MP-specific, VDT-specific	MP-specific sensitivity group: N = 45, m.a = 45.7, f.g = 62%. General sensitivity group: N = 71, m.a = 51.6 f.g = 82%.	Report of symptoms attributed to: 1. MP use only, 2. VDT use only or 3several types of electrical equipment.	N.R/E.	Subjective report.
Mohler et al., 2010 (Switzerland) [86].	Cross-sectional	Randomly selected sample from general population (N = 1212).	General	N = 253.	Self-reported electrohypersensitivity or report of adverse health effects attributed to EMF.	Consumption of sleeping pills, night shift workers, severe disability.	Subjective report.
Nordin et al., 2010 (Sweden) [87].	Cross-sectional	Voluntary participation of IEI subjects after description of the study in a local and a national newspaper.	General	N = 2, a.r = 18-69.	Report of being intolerant to EMF.	Pregnancy.	Subjective report.
Röösli et al., 2010 (Switzerland) [88].	Cross-sectional	Randomly selected sample from general population (N = 1122).	General	N = 130, a.r = 30-60, f.g = 72.3%.	Self-reported hypersensitivity to EMF.	N.R/E.	Subjective report.

*Although the studies of Carlsson et al. [7] and Österberg et al. [45] are based on the same sample [46], they have some differences in terms of inclusion criteria and/or identification methods. This was the case also for the studies of Mohler et al. [86] and Röösli et al. [88].

Abbreviations: N.R., Not reported; N.R/E, Not reported or employed. FTL, Fluorescent tube light; EMF, Electromagnetic fields; IEI-EMF, Idiopathic environmental intolerance attributed to EMF; m.a, Mean age; a.r, Age range; f.g, Female gender distribution; y.o; Years old; MP, Mobile phone(s); VDT, Video display terminal: VDU, Video display units.

### Review Process

The literature search was performed by the first author and the evaluation of inclusion criteria by CB, IVK and GJR, with uncertainties resolved through consultation among all the authors. The initial screening was based on the titles and/or abstracts. Next, the hard copies of the potentially eligible publications were examined to assess whether they met the inclusion criteria.

## Results

### Search results

**Figure 1:**
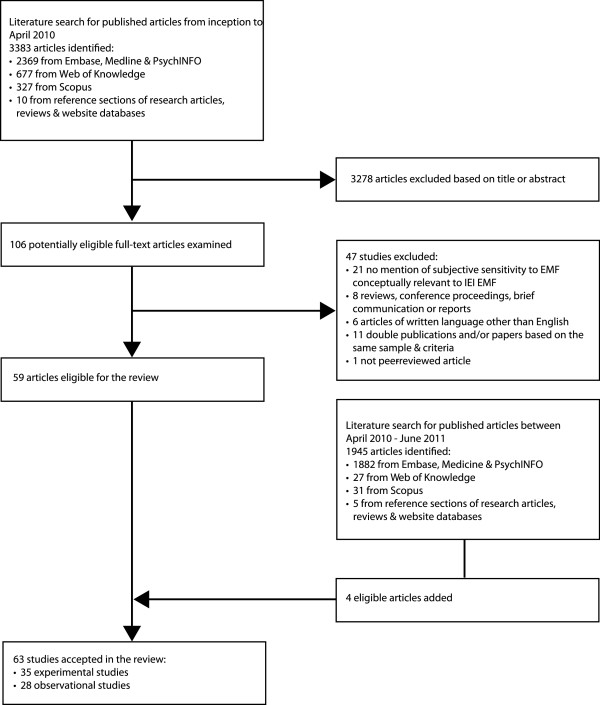
**Flow diagram outlining the study selection process**.

Figure 1 illustrates the literature search process. We examined 5328 citations in total and identified 35 experimental and 28 observational studies that met our inclusion criteria.

### Study characteristics

**Figure 2:**
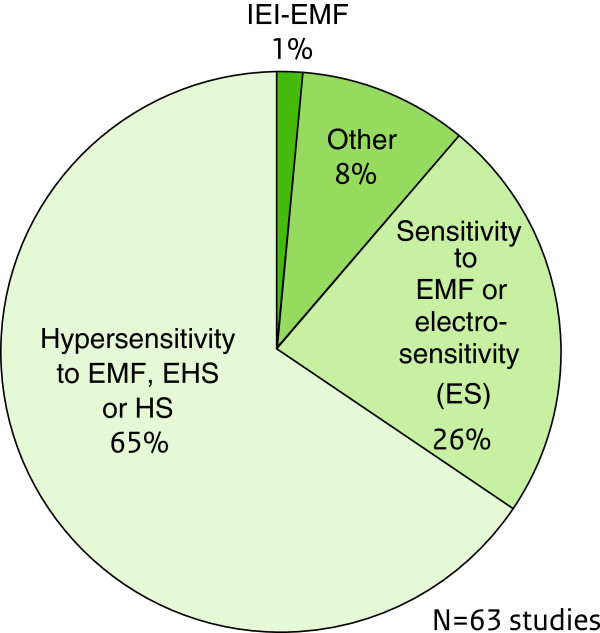
**Distribution (%) of terms used to describe IEI-EMF in the reviewed literature.** Abbreviations: IEI-EMF, Idiopathic environmental intolerance attributed to EMF; EHS, Electrohypersensitivity; HS, Hypersensitivity.

When reported, sample sizes of subjects with IEI-EMF ranged between 1 to 100 in the experimental studies and from 2 to 2748 in the observational studies. The percentage of female participants (exempting case-studies) ranged between 0 to 81.3% and 50% to 100% respectively. In all studies, the reported mean age of IEI-EMF individuals varied between 26.1 and 55.5 years. IEI-EMF triggered by several different EMF sources (“general”) was the sensitivity type of primary focus in the included investigations (n = 48), while 14 studies concentrated exclusively on “source-specific” IEI-EMF and three on both “general” and “source-specific” IEI-EMF. There was a variety of synonyms of IEI-EMF in the literature such as "hypersensitivity (HS) to EMF", "electromagnetic Hypersensitivity (EHS)", "electrohypersensitivity", "environmental annoyance attributed to EMF", "electromagnetic distress syndrome" and "environmental illness". “Hypersensitivity to EMF” (and its variants) was by far the most frequently used definition/descriptive term (Figure 2). In 35 studies the case definition procedure was solely based on the subjective report of the respondents. In 28 studies it was mentioned that objective assessment (e.g. medical and/or psychological assessment) was additionally taken into account.

The principal method of sample recruitment was via study description in advertisements and/or local or national media (22 studies). The vast majority of the reviewed studies were conducted in Europe (58 studies).

### Experimental studies

The major inclusion criteria used by experimental studies to identify individuals with IEI-EMF were:

• Attribution of NSPS to either various or specific sources of EMF (being reported 13 times).

• Self-reported IEI-EMF (or synonymous terms) (n = 14).

• Experience of symptoms during or soon (from 20 minutes to 24 hours) after the individual perception or actual presence or use of an EMF exposure source (n = 10).

• High score on a symptom scale (n = 6).

In addition, two studies used limitation in daily functioning of the individual due to the attributed health effects as an inclusion criterion.

The main exclusion criterion was the existence of a medical and/or psychiatric or psychological condition that could account for the reported health complaints (n = 20).

Other exclusion criteria included undergoing treatment for somatic or psychiatric conditions (n = 8), pregnancy (n = 5), history of severe injuries (n = 3) and regular smoking (n = 2).

In 16 studies the case definition procedure did not only rely on subjective report, but also on medical and/or psychiatric and/or psychological examination. In eight studies, the sample recruitment was based on participants who were already referred or registered to a health care institution (such as a university hospital) for their health complaints. All extracted data from the experimental studies are presented in Figure 2.

### Observational Studies

The major inclusion criteria used by observational studies to identify individuals with IEI-EMF were:

• Self-reported IEI-EMF (or synonymous terms) (n = 16).

• Attribution of NSPS to either various or specific EMF sources (n = 12).

• Experience of symptoms during or soon (from 20 minutes to 24 hours) after the individual perception or actual presence or use of an EMF exposure source (n = 3).

• Limitation in daily functioning of the individual due to the attributed health effects (n = 2).

The main exclusion criteria were a medical and/or psychiatric or psychological condition that could account for the reported health complaints and undergoing treatment for somatic or psychiatric condition (n = 4).

Eleven studies included medical and/or psychiatric and/or psychological examination to assess whether a pathological condition was responsible for patients’ complaints. In nine studies the sample was based on participants who were already referred or registered to health care institutions for their complaints. All extracted data from the observational studies are listed in Table 3.

**Figure 3:**
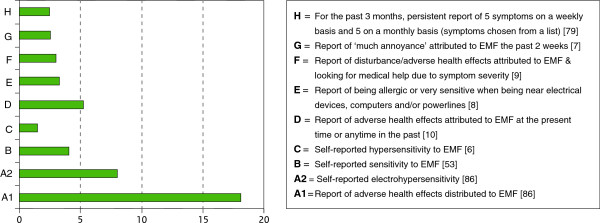
**Prevalence (%) of IEI-EMF based on the identifying criteria employed by population-based observational studies**.

The prevalence of IEI-EMF in randomly selected samples of population-based epidemiological studies varied and seemed to be influenced by the number and degree of strictness of the applied identification criteria. This is illustrated in Figure 3. These differences could also be due to the population under study, year of study and sample stratification (e.g. age range).

## Discussion

The present systematic review based on an extensive literature search, summarized the case definition criteria and methods that have been used in the published literature to date for the identification of subjects with IEI-EMF.

It is noteworthy that only 1% of the reviewed studies used the term “IEI-EMF” as a descriptive term, despite the fact that it has been proposed by WHO since 2005 [5]. Sixty-five percent of the studies used the description “Hypersensitivity to EMF” which seems to be mainly characterized by the following aspects: Self-reported sensitivity to one or more sources of EMF, attribution of NSPS to either several or specific EMF sources (such as mobile phones and VDUs), experience of symptoms during or soon after (from 20 minutes to 24 hours) the individual perception or actual presence or use of an EMF source and absence of a (psycho)pathological condition accounting for the reported health complaints. In the majority of the studies the case definition procedure was based exclusively on self-report. In a smaller number of investigations, medical and/or psychiatric and/or psychological assessment was included.

In most of these studies participants were recruited from registries to a health care institution for their symptoms and for whom medical data were available. Although there were no important differences between observational and experimental studies in the most frequently employed criteria, experimental studies used a larger number of criteria per investigation compared to observational studies. Moreover, the demographic profile of the recruited individuals with IEI-EMF in terms of age and gender was quite consistent; the frequency of female gender and age over 40 years were considerably higher in most of the studies.

Despite previous attempts to bring order to this field [6,53,70], as it appears in the literature, IEI-EMF is still predominantly a self-reported sensitivity without a widely accepted and validated case definition tool. This could be due to the absence of a bioelectromagnetic mechanism [17] or because of the varying patterns regarding the symptom type, frequency and severity [6,41]. The other way around could also be the case: The lack of validated case definition criteria could have hindered the identification of homogeneous patient groups and consequently the recognition of symptom profiles and a physiologic mechanism. Furthermore, the application of very broad criteria could dilute the power of the studies and make difficult the detection of those individuals that really suffer from IEI-EMF. For example, although “Attribution” of NSPS to EMF could be considered as a first indication of suffering from IEI-EMF, it is questionable whether it comprises a sufficient identifying criterion when used alone.

### Possible subgroups

Several subdivisions may exist within IEI-EMF that may be of relevance to clinicians and researchers.

One such division is that between patients for whom an alternative diagnosis exists, which might account for their symptoms and those for whom it does not. The absence of screening for pathological conditions which might underlie the symptoms reported by participants in many studies was notable. Previous studies have identified occasionally high levels of other diagnoses in such patients, such as somatoform and anxiety disorders which might account for their ill-health [89,90]. Including these individuals in the same sample as those for whom there is no clear explanation for their symptoms may reduce our ability to identify causal factors for IEI-EMF.

An additional distinction that we may need to take into account is between patients who attribute symptoms to short-term exposure to EMF and those for whom longer-term exposure is relevant. Furthermore, it remains unclear whether generalized and source-specific IEI-EMF should be assessed separately or not. Exposure from far-field sources such as high-voltage overhead powerlines and mobile phone base stations is mostly continuous and people often perceive it as less controllable compared to near-field sources such as mobile phones [10] but there is still no convincing evidence for source-specific sensitivities [13]. As differences may exist between IEI-EMF patients in terms of their psychological and health-related characteristics, division into subgroups for the purposes of research may be of use [22,23]. Perhaps the most complicated issue is to figure out whether self-reported-NSPS and objectively assessed physiologic reactions are preceded by events of the relevant (EMF) exposures, distinguishable from other random exposure events experienced during the day. Use of a prediction model based on modelled exposure from various sources [91,92] could be a solution; however it is questionable whether and how it could be systematically incorporated in a case definition tool.

Table 4 illustrates a number of proposed aspects for IEI-EMF, based on a synthesis of the existing identifying criteria in the reviewed literature. Considering the fact that the reported symptoms are quite common in the general population and also the lack of symptom patterns [6,53] and etiology, the only parameter that clearly distinguishes sensitive from control individuals is the causal attribution of symptomatology to EMF exposure. Therefore, the attribution of health outcomes and self-reported sensitivity to EMF inevitably constitute, at the moment, the cornerstone of IEI-EMF case definition in research and clinical practice. Additional aspects such as medical examination/history would elucidate whether the reported health outcomes can be explained by underlying pathology. Cognitive and behavioral aspects could be complementarily included in the case definition, since evidence on their role in IEI-EMF is promising [18] but not yet established. Moreover, taking into account potentially harmful environmental agents other than EMF would be an important addition for research.

**Table 4:** Proposed case definition aspects for IEI-EMF

		
**Dimensions of IEI-EMF**	**Case definition assessment/identification of IEI-EMF**	
	** *Research* **	** *Clinical practice* **
Health effects	- Subjective report of symptoms/physiologic reactions.	- Subjective report of symptoms/physiologic reactions.
	- The possibility that a known medical or psychiatric condition is the cause of the reported health complaints should be excluded with the use of standardized interview and patient history.	- The possibility that a known medical or psychiatric condition is the cause of the reported health complaints should be excluded after thorough physical and psychiatric examination and detailed patient history.
	- Current status of residential and occupational exposure to harmful environmental agents that could be related to the reported complaints (other than non-ionizing EMF).	
Triggering factors	- Attribution of NSPS or other physiologic reaction(s) to either all/several EMF sources (General IEI-EMF) or one specific EMF source (such as VDU, MP or FTL)and/or	
	- Subjective report of being sensitive to specific or various EMF sources.	
Cognition & behavior (optional)	- Symptoms occur during or after the individual perception or actual exposure, presence or use of an EMF source.	
	- Regular avoidance behavior towards EMF source(s) due to the fear of a negative impact of EMF on health.	

This is the first time that a systematic review is conducted on definitions and identifying criteria for IEI-EMF. Given the large number of included articles, it is unlikely that any missing (or even excluded) studies would alter the results or increase any publication bias, especially since the aim of the current paper was not to assess etiologic associations.

It is a challenge how all the different case definition parameters for IEI-EMF can be concisely embodied in one international operational tool which could be used in research and clinical practice, and how this instrument could be adjusted to the possible cultural differences (e.g. in terms of wording/phrasing questions on health outcomes). Nevertheless, without the harmonization of the conceptual framework and validation of identifying criteria, the value of the case definition standards for IEI-EMF will remain insufficient and possibly unreliable. Apart from research, this has an important impact also in primary care; physicians, who are often the first to be contacted by the sufferers, are usually not adequately informed about IEI-EMF, which can affect the patient-doctor interaction and the management of the patient [26].

In order to properly construct an operational tool, a proposed two-phase approach can be briefly described as follows: In the first phase, a case definition and case selection tool should be developed, taking into account sources such as the published literature, expert opinions (e.g. based on a Delphi procedure [93]) and information on IEI-EMF patient characteristics from available datasets/ongoing research. At this stage, EMF measurements or provocation tests should not be a priority since a provocation study will only have added value after the formulation of a proper case definition and participant selection. Additionally, if the aim of a “case selection tool” is to routinely test cases where symptoms occur without a clear underlying pathology, then that tool should be concise, inexpensive and easy to implement, such as a short questionnaire or checklist. In the second phase, the case definition tool should be validated in terms of practical usability and the ability to differentiate between subgroups of IEI-EMF and patients with other conditions (e.g. chronic fatigue) who report similar symptoms. Based on the findings, the requirements for a follow up study could be outlined.

## Conclusions

IEI-EMF is a poorly defined sensitivity. Heterogeneity and ambiguity of the existing definitions and criteria for IEI-EMF show the necessity to develop uniform criteria that will be applicable both in research and clinical practice. Broader criteria identified in the published literature such as attribution of NSPS to EMF and subjective report of being EMF sensitive could be used as a working definition for IEI-EMF which will serve as a basis for the development of a case selection tool. However, further optimization is required, testing its reliability and validity in several different patient groups, leading to an international multidisciplinary protocol with the active involvement of health care providers. This could also be a stepping stone for the harmonization of concepts and case definition for the broader condition of IEI.

## Abbreviations

EMF: Electromagnetic Fields; EHS: Electrohypersensitivity; HS: Hypersensitivity; IEI: Idiopathic Environmental Intolerance; IEI-EMF: Idiopathic Environmental Intolerance attributed to Electromagnetic Fields; MP: Mobile Phone(s); NSPS: Non-Specific Physical Symptoms; VDU: Video Display Units; VDT: Video Display Terminals.

## Competing interests

GJR has previously been funded by the UK Mobile Telecommunications and Health Research programme ( http://www.MTHR.org.uk) and has acted as an expert witness for the Diocese of Norwich relating to the installation of Wifi on Church property. EL chairs the Science Forum of the Dutch Expertise Platform for Electromagnetic Fields ( http://www.kennisplatform.nl/).

## Authors’ contributions

All the authors participate in the international multidisciplinary project INCORPORATE of the National Institute for Public Health and the Environment (RIVM). CB carried out the literature search, drafted the manuscript and incorporated input from all the rest authors in the manuscript. IVK and GJR conceived and coordinated the study and provided critical comments on the manuscript. EL provided critical comments on the manuscript. All authors have read and approved the final version of the manuscript.

## Pre-publication history

The pre-publication history for this paper can be accessed here:

http://www.biomedcentral.com/1471-2458/12/643/prepub

## References

[B1] Randolph TG Human ecology and susceptibility to the chemical environment 1962 Springfield (IL): Charles Thomas

[B2] Das-Munshi J Rubin GJ Wessely S Multiple chemical sensitivities: A systematic review of provocation studies J Allergy Clin Immunol 2006 118 1257 1264 10.1016/j.jaci.2006.07.046 17137865

[B3] Reed-Gibson P Elms ANM Ruding LA Perceived Treatment Efficacy for Conventional and Alternative Therapies Reported by Persons with Multiple Chemical Sensitivity Environ Health Perspect 2003 111 1498 1504 10.1289/ehp.5936 12948890 PMC1241653

[B4] Röösli M Moser M Baldinini Y Meier M Braun-Fahrländer C Symptoms of ill health ascribed to electromagnetic field exposure—A questionnaire survey Int J Hygiene Environ Health 2004 207 141 150 10.1078/1438-4639-00269 15031956

[B5] WHO Fact Sheet No. 296: Electromagnetic fields and public health 2005 World Health Organization http://www.emfandhealth.com/WHO_EMSensitivity.pdf

[B6] Hillert L Berglind N Arnetz BB Bellander T Prevalence of self-reported hypersensitivity to electric or magnetic fields in a population-based questionnaire survey Scand J Work Environ Health 2002 28 33 41 10.5271/sjweh.644 11871850

[B7] Carlsson F Karlson B Orbaek P Österberg K Östergren PO Prevalence of annoyance attributed to electrical equipment and smells in a Swedish population, and relationship with subjective health and daily functioning Public Health 2005 119 568 577 10.1016/j.puhe.2004.07.011 15925670

[B8] Levallois P Neutra R Lee G Hristova L Study of self-reported hypersensitivity to electromagnetic fields in California Environ Health Perspect 2002 110 619 623 10.1289/ehp.02110s4619 12194896 PMC1241215

[B9] Schröttner J Leitgeb N Sensitivity to electricity - temporal changes in Austria BMC Publ Health 2008 8 310 10.1186/1471-2458-8-310 PMC2562386 18789137

[B10] Schreier N Huss A Röösli M The prevalence of symptoms attributed to electromagnetic field exposure: a cross-sectional representative survey in Switzerland Soz Praventivmed 2006 51 202 209 10.1007/s00038-006-5061-2 17193782

[B11] Tseng MM Lin YP Cheng TJ Prevalence and Psychiatric Co-Morbidity of Self-Reported Electromagnetic Field Sensitivity in Taiwan: A Population-Based Study Epidemiology 2008 19 s108 s109 10.1016/j.jfma.2011.08.005 21982467

[B12] Levallois P Hypersensitivity of human subjects to environmental electric and magnetic field exposure: A review of the literature Environ Health Perspect 2002 110 613 618 12194895 10.1289/ehp.02110s4613 PMC1241214

[B13] Rubin GJ Das-Munshi J Wessely S Electromagnetic hypersensitivity: A systematic review of provocation studies Psychosom Med 2005 67 224 232 10.1097/01.psy.0000155664.13300.64 15784787

[B14] Röösli M Radiofrequency electromagnetic field exposure and non-specific symptoms of ill health: a systematic review Environ Res 2008 107 277 287 10.1016/j.envres.2008.02.003 18359015

[B15] Rubin GJ Nieto-Hernandez R Wessely S Idiopathic environmental intolerance attributed to electromagnetic fields (formerly 'electromagnetic hypersensitivity'): An updated systematic review of provocation studies Bioelectromagnetics 2009 31 1 11 19681059 10.1002/bem.20536

[B16] Röösli M Frei P Mohler E Hug K Systematic review on the health effects of exposure to radiofrequency electromagnetic fields from mobile phone base stations Bull World Health Organ 2010 88 887 896 10.2471/BLT.09.071852 21124713 PMC2995180

[B17] Rubin GJ Hillert L Nieto-Hernandez R Van Rongen E Oftedal G Do people with idiopathic environmental intolerance attributed to electromagnetic fields display physiological effects when exposed to electromagnetic fields? A systematic review of provocation studies Bioelectromagnetics 2011 32 593 609 10.1002/bem.20690 21769898

[B18] Rubin GJ Das Munshi J, Wessely S: A systematic review of treatments for electromagnetic hypersensitivity Psychother Psychosom 2006 75 12 18 10.1159/000089222 16361870

[B19] Österberg K Persson R Karlson B Carlsson EF Orbaek P Personality, mental distress, and subjective health complaints among persons with environmental annoyance Hum Exp Toxicol 2007 26 231 241 10.1177/0960327107070575 17439926

[B20] Landgrebe M Frick U Hauser S Langguth B Rosner R Hajak G Eichhammer P Cognitive and neurobiological alterations in electromagnetic hypersensitive patients: Results of a case–control study Psychol Med 2008 38 1781 1791 10.1017/S0033291708003097 18366821

[B21] Persson R Carlsson EF Österberg K Orbaek P Karlson B A two-week monitoring of self-reported arousal, worry and attribution among persons with annoyance attributed to electrical equipment and smells Scand J Psychol 2008 49 345 356 10.1111/j.1467-9450.2008.00660.x 18466187

[B22] Rubin GJ Cleare AJ Wessely S Psychological factors associated with self-reported sensitivity to mobile phones J Psychosom Res 2008 64 1 9 10.1016/j.jpsychores.2007.05.006 18157992

[B23] Johansson A Nordin S Heiden M Sandström M Symptoms, personality traits, and stress in people with mobile phone-related symptoms and electromagnetic hypersensitivity J Psychosom Res 2010 68 37 45 10.1016/j.jpsychores.2009.06.009 20004299

[B24] Baliatsas C Van Kamp I Kelfkens G Schipper M Bolte J Yzermans J Lebret E Non-specific physical symptoms in relation to actual and perceived proximity to mobile phone base stations and powerlines BMC Publ Health 2011 11 421 10.1186/1471-2458-11-421 PMC3118249 21631930

[B25] Huss A Röösli M Consultations in primary care for symptoms attributed to electromagnetic fields – a survey among general practitioners BMC Publ Health 2006 6 267 10.1186/1471-2458-6-267 PMC1635563 17074080

[B26] Berg-Beckhoff G Heyer K Kowall B Breckenkamp J Razum O The views of primary care physicians on health risks from electromagnetic fields Dtsch Arztebl Int 2010 107 817 823 21151417 10.3238/arztebl.2010.0817 PMC2999946

[B27] Salomon D Medical practitioners and electromagnetic fields (EMF): Testing their concern Comptes Rendus Physique 2010 11 636 640 10.1016/j.crhy.2011.01.001

[B28] Rea WJ Pan Y Fenyves EJ Sujisawa I Samadi N Ross GH Electromagnetic field sensitivity J Bioelectricity 1991 10 241 256

[B29] Hamnerius Y Agrup G Galt S Nilsson R Sandblom J Lindgren R Double-blind provocation study of hypersensitivity reactions associated with exposure from VDUs. Preliminary short version R Swed Acad Sci Rep 1993 2 67 72

[B30] Arnetz BB Berg M Anderzen I Lundeberg T Haker E A nonconventional approach to the treatment of ‘environmental illness’ J Occup Environ Med 1995 37 838 844 10.1097/00043764-199507000-00013 7552468

[B31] Andersson B Berg M Arnetz BB Melin L Langlet I Liden SA Cognitive-behavioral treatment of patients suffering from ’electric hypersensitivity’: subjective effects and reactions in a double-blind provocation study J Occup Environ Med 1996 38 752 758 10.1097/00043764-199608000-00009 8863199

[B32] Bertoft G Patient reactions to some electromagnetic fields from dental chair and unit: a pilot study Swed Dent J 1996 20 107 112 8957136

[B33] Toomingas A Provocation of the electromagnetic distress syndrome Scand J Work Environ Health 1997 22 457 458 9000314 10.5271/sjweh.168

[B34] Sandström M Lyskov E Berglund A Medvedev S Hansson-Mild K Neurophysiological Effects of Flickering Light in Patients with Perceived Electrical Hypersensitivity J Occup Environ Med 1997 39 15 22 10.1097/00043764-199701000-00006 9029427

[B35] Hillert L Hedman BK Dolling BF Arnetz BB Cognitive behavioural therapy for patients with electric sensitivity – A multidisciplinary approach in a controlled study Psychother Psychosom 1998 67 302 310 10.1159/000012295 9817951

[B36] Trimmel M Schweiger E Effects of an ELF (50 Hz, 1 mT) electromagnetic field (EMF) on concentration in visual attention, perception and memory including effects of EMF sensitivity Toxicol Lett 1998 96-97 377 382 9820691 10.1016/s0378-4274(98)00096-4

[B37] Flodin U Seneby A Tegenfeldt C Provocation of electric hypersensitivity under everyday conditions Scand J Work Environ Health 2000 26 93 98 10.5271/sjweh.517 10817373

[B38] Lonne-Rahm S Andersson B Melin L Schultzberg M Arnetz B Berg M Provocation with stress and electricity of patients with ’sensitivity to electricity. J Occup Environ Med 2000 42 512 516 10.1097/00043764-200005000-00009 10824304

[B39] Hillert L Kolmodin-Hedman B Eneroth P Arnetz BB The effect of supplementary antioxidant therapy in patients who report hypersensitivity to electricity: a randomized controlled trial Medscape General Medicine 2001 3 11 11549960

[B40] Lyskov E Sandström M Hansson-Mild K Provocation study of persons with perceived electrical hypersensitivity and controls using magnetic field exposure and recording of electrophysiological characteristics Bioelectromagnetics 2001 22 457 462 10.1002/bem.73 11568930

[B41] Hietanen M Hamalainen AM Husman T Hypersensitivity symptoms associated with exposure to cellular telephones: no causal link Bioelectromagnetics 2002 23 264 270 10.1002/bem.10016 11948605

[B42] Hillert L Savlin P Levy BA Heidenberg A Kolmodin-Hedman B Environmental illness – Effectiveness of a salutogenic group-intervention programme Scand J Public Health 2002 30 166 175 10.1080/14034940210133852 12357983

[B43] Mueller CH Krueger H Schierz C Project NEMESIS: perception of a 50 Hz electric and magnetic field at low intensities (laboratory experiment) Bioelectromagnetics 2002 23 26 36 10.1002/bem.95 11793403

[B44] Leitgeb N Schröttner J Electrosensibility and electromagnetic hypersensitivity Bioelectromagnetics 2003 24 387 394 10.1002/bem.10138 12929157

[B45] Österberg K Persson R Karlson B Orbæk P Annoyance and performance in three groups of environmentally intolerant subjects during experimental challenge with chemical odors Scand J Work Environ Health 2004 6 486 496 15633599

[B46] Ostergren PO Merlo J Lindström M Rosvall M Kahn FA Lithman T Hälsoförhållanden i Skåne. Folkhälsoenkät Skåne 2000 (Report in Swedish) 2001 Region Skåne: Kommunförbundet Skåne och Skåne läns allmänna Försäkringskassa

[B47] Belyaev IY Hillert L Protopopova M Tamm C Malmgren LOG Persson BRR Selivanova G Harms-Ringdahl M 915 MHz microwaves and 50 Hz magnetic field affect chromatin conformation and 53BP1 foci in human lymphocytes from hypersensitive and healthy persons Bioelectromagnetics 2005 26 173 184 10.1002/bem.20103 15768430

[B48] Frick U Kharraz A Hauser S Wiegand R Rehm J Von Kovatsits U Eichhammer P Comparison perception of singular transcranial magnetic stimuli by subjectively electrosensitive subjects and general population controls Bioelectromagnetics 2005 26 287 298 10.1002/bem.20085 15832334

[B49] Wenzel F Reissenweber J David E Cutaneous microcirculation is not altered by a weak 50 Hz magnetic field Biomed Tech 2005 50 14 18 10.1515/BMT.2005.003 15792196

[B50] Regel SJ Negovetic S Röösli M Berdinas V Schuderer J Huss A Lott U Kuster N Achermann P UMTS base stationlike exposure, well-being, and cognitive performance Environ Health Perspect 2006 114 1270 1275 10.1289/ehp.8934 16882538 PMC1552030

[B51] Rubin GJ Hahn G Everitt B Cleare AJ Wessely S Are some people sensitive to mobile phone signals? A within participants, double-blind, randomised provocation study Br Med J 2006 332 886 889 10.1136/bmj.38765.519850.55 16520326 PMC1440612

[B52] Eltiti S Wallace D Ridgewell A Zougkou K Russo R Sepulveda F Mirshekar-Syahkal D Rasor P Deeble R Fox E Does short-term exposure to mobile phone base station signals increase symptoms in individuals who report sensitivity to electromagnetic fields? A double- blind randomized provocation study Environ Health Perspect 2007 115 1603 1608 10.1289/ehp.10286 18007992 PMC2072835

[B53] Eltiti S Wallace D Zougkou K Russo R Joseph S Rasor P Fox E Development and evaluation of the electromagnetic hypersensitivity questionnaire Bioelectromagnetics 2007 28 137 151 10.1002/bem.20279 17013888

[B54] Schröttner J Leitgeb N Hillert L Investigation of Electric Current Perception Thershold of Different EHS Groups Bioelectromagnetics 2007 28 208 213 10.1002/bem.20294 17080457

[B55] Bamiou DE Ceranic B Cox R Watt H Chadwick P Luxon LM Mobile telephone use effects on peripheral audiovestibular function: A case- control study Bioelectromagnetics 2008 29 108 117 10.1002/bem.20369 17929266

[B56] Hillert L Akerstedt T Lowden A Wiholm C Kuster N Ebert S Boutry C Moffat SD Berg M Arnetz BB The effects of 884 MHz GSM wireless communication signals on headache and other symptoms: An experimental provocation study Bioelectromagnetics 2008 29 185 196 10.1002/bem.20379 18044740

[B57] Kwon MS Koivisto M Laine M Hamalainen H Perception of the electromagnetic field emitted by a mobile phone Bioelectromagnetics 2008 29 154 159 10.1002/bem.20375 18027840

[B58] Landgrebe M Barta W Rosengarth K Frick U Hauser S Langguth B Rutschmann R Greenlee MW Hajak G Eichhammer P Neuronal correlates of symptom formation in functional somatic syndromes: A fMRI study NeuroImage 2008 41 1336 1344 10.1016/j.neuroimage.2008.04.171 18499479

[B59] Frick U Mayer M Hauser S Binder H Rosner R Eichhammer P Entwicklung eines deutschsprachigen Messinstruments für “Elektrosmog-Beschwerden” (in German) Umweltmedizin in Forschung & Praxis 2006 11 11 22

[B60] Leitgeb N Schröttner J Cech R Kerbl R EMF-protection sleep study near mobile phone base stations Somnologie 2008 12 234 243 10.1007/s11818-008-0353-9

[B61] Fahrenberg J Die Freiburger Beschwerdenliste (FBL) (in German) Z Klin Psychol 1975 4 79 100

[B62] Buysse DJ Reynolds CF Monk TH Berman SR Kupfer DJ The Pittsburgh Sleep Quality Index: a new instrument for psychiatric practice and research Psychiatry Res 1989 28 193 213 10.1016/0165-1781(89)90047-4 2748771

[B63] Augner C Florian M Pauser G Oberfeld G Hacker GW GSM base stations: Short-term effects on well-being Bioelectromagnetics 2009 30 73 80 10.1002/bem.20447 18803247

[B64] Furubayashi T Ushiyama A Terao Y Mizuno Y Shirasawa K Pongpaibool P Simba AY Wake K Nishikawa M Miyawaki K Yasuda A Uchiyama M Yamashita HK Masuda H Hirota S Takahashi M Okano T Inomata-Terada S Sokejima S Maruyama E Watanabe S Taki M Ohkubo C Ugawa Y Effects of short-term W-CDMA mobile phone base station exposure on women with or without mobile phone related symptoms Bioelectromagnetics 2009 30 100 113 10.1002/bem.20446 18780296

[B65] Nam KC Lee JH Noh HW Cha EJ Kim NH Kim DW Hypersensitivity to RF fields emitted from CDMA cellular phones: A provocation study Bioelectromagnetics 2009 30 641 650 10.1002/bem.20518 19551766

[B66] Szemerszky R Koteles F Lihi R Bardos G Polluted places or polluted minds? An experimental sham-exposure study on background psychological factors of symptom formation in ‘Idiophatic Environmental Intolerance attributed to electromagnetic fields’ Int J Hyg Environ Health 2010 213 387 394 10.1016/j.ijheh.2010.05.001 20538519

[B67] Nieto-Hernandez R Williams J Cleare AJ Landau S Wessely S Rubin GJ Can exposure to a terrestrial trunked radio (TETRA)-like signal cause symptoms? A randomized double-blind provocation study Occup Environ Med 2011 68 339 344 10.1136/oem.2010.055889 20864469

[B68] Bergdahl J Tillberg A Stenman E Odontologic survey of referred patients with symptoms allegedly caused by electricity or visual display units Acta Odontol Scand 1998 56 303 307 10.1080/000163598428491 9860100

[B69] Hocking B Preliminary report: symptoms associated with mobile phone use Occup Med 1998 48 357 360 10.1093/occmed/48.6.357 10024730

[B70] Hillert L Hedman BK Söderman E Arnetz BB Hypersensitivity to electricity: Working definition and additional characterization of the syndrome J Psychosom Res 1999 47 429 438 10.1016/S0022-3999(99)00048-3 10624841

[B71] Stockenius S Brugger P Perceived electrosensitivity and magical ideation Percept Mot Skills 2000 90 899 900 10.2466/pms.2000.90.3.899 10883774

[B72] Bergdahl J Bergdahl M Environmental illness: evaluation of salivary flow, symptoms, diseases, medications, and psychological factors Acta Odontol Scand 2001 59 104 110 10.1080/000163501750157270 11370747

[B73] Hillert L Flato S Georgellis A Arnetz BB Kolmodin-Hedman B Environmental illness: Fatigue and cholinesterase activity in patients reporting hypersensitivity to electricity Environ Res 2001 85 200 206 10.1006/enrs.2000.4225 11237508

[B74] Lyskov E Sandström M Hansson Mild K: Neurophysiological study of patients with perceived electrical hypersensitivity Int J Psychophysiol 2001 42 233 241 10.1016/S0167-8760(01)00141-6 11812390

[B75] Stenberg B Bergdahl J Edvardsson B Eriksson N Lindén G Widman L Medical and social prognosis for patients with perceived hypersensitivity to electricity and skin symptoms related to the use of visual display terminals Scand J Work Environ Health 2002 28 349 357 10.5271/sjweh.685 12432989

[B76] Sandström M Lyskov E Hörnsten R Hansson-Mild, Wiklund U, Rask P, Klucharev V, Stenberg B, Bjerle P: ECG monitoring in patients with perceived electrical hypersensitivity Int J Psychophysiol 2003 49 227 235 10.1016/S0167-8760(03)00145-4 14507441

[B77] Bergdahl J Stenberg B Eriksson N Lindén G Widman L Coping and self-image in patients with visual display terminal-related skin symptoms andperceived hypersensitivity to electricity Int Arch Occup Environ Health 2004 77 538 542 10.1007/s00420-004-0546-x 15538619

[B78] Bergdahl J Marell L Bergdahl M Perris H Psychobiological personality dimensions in two environmental-illness patient groups Clin Oral Investig 2005 9 251 256 10.1007/s00784-005-0015-2 16215748

[B79] Eriksson NM Stenberg BG Baseline prevalence of symptoms related to indoor environment Scand J Public Health 2006 34 387 396 10.1080/14034940500228281 16861189

[B80] Schüz J Petters C Egle UT Jansen B Kimbel R Letzel S Nix W Schmidt LG Vollrath L The “Mainzer EMF-Wachhund”: results from a watchdog project on self-reported health complaints attributed to exposure to electromagnetic fields Bioelectromagnetics 2006 27 280 287 10.1002/bem.20212 16511876

[B81] Landgrebe M Hauser S Langguth B Frick U Hajak G Eichhammer P Altered cortical excitability in subjectively electrosensitive patients: results of a pilot study J Psychosom Res 2007 62 283 288 10.1016/j.jpsychores.2006.11.007 17324677

[B82] Hardell L Carlberg M Söderqvist F Hardell K Björnfoth H van Bavel B Lindström G Increased concentrations of certain persistent organic pollutants in subjects with self-reported electromagnetic hypersensitivity–a pilot study Electromagn Biol Med 2008 27 197 203 10.1080/15368370802089053 18568937

[B83] Lidmark AM Wikmans T Are they really sick? A report on persons who are electrosensitive and/or injured by dental material in Sweden Journal of Orthomolecular Medicine 2008 23 153 160

[B84] Dahmen N Ghezel-Ahmadi D Engel A Blood Laboratory Findings in Patients Suffering From Self-Perceived Electromagnetic Hypersensitivity (EHS) Bioelectromagnetics 2009 30 299 306 10.1002/bem.20486 19259984

[B85] Frick U Rehm J Eichhammer P Risk perception, somatization, and self report of complaints related to electromagnetic fields—a randomized survey study Int J Hyg Environ Health 2002 205 353 360 10.1078/1438-4639-00170 12173533

[B86] Mohler E Frei P Fahrländer CB Fröhlich J Neubauer G Röösli M Effects of Everyday Radiofrequency Electromagnetic-Field Exposure on Sleep Quality: A Cross-Sectional Study Radiat Res 2010 174 347 356 10.1667/RR2153.1 20726726

[B87] Nordin M Andersson L Nordin S Coping strategies, social support and responsibility in chemical Intolerance J Clin Nurs 2010 19 2162 2173 10.1111/j.1365-2702.2010.03264.x 20659196

[B88] Röösli M Mohler E Frei P Sense and sensibility in the context of radiofrequency electromagnetic field exposure Comptes Rendus Physique 2010 11 576 584 10.1016/j.crhy.2010.10.007

[B89] Bornschein S Hausteiner C Zilker T Förstl H Psychiatric and somatic disorders and multiple chemical sensitivity (MCS) in 264 environmental patients Psychol Med 2002 32 1387 1394 12455937 10.1017/s0033291702006554

[B90] Bailer J Witthöft M Paul C Bayerl C Rist F Evidence for overlap between idiopathic environmental intolerance and somatoform disorders Psychosom Med 2005 67 921 929 10.1097/01.psy.0000174170.66109.b7 16314597

[B91] Frei P Mohler E Bürgi A Fröhlich J Neubauer G Braun-Fahrländer C Röösli M A prediction model for personal radio frequency electromagnetic field exposure Sc of the Tot Env 2009 408 102 108 10.1016/j.scitotenv.2009.09.023 19819523

[B92] Frei P Mohler E Bürgi A Fröhlich J Neubauer G Braun-Fahrländer C Röösli M Classification of personal exposure to radio frequency electromagnetic fields (RF-EMF) for epidemiological research: Evaluation of different exposure assessment methods Environ Int 2010 36 714 720 10.1016/j.envint.2010.05.005 20538340

[B93] Hasson F Keeney S McKenna H Research guidelines for the Delphi survey technique J Adv Nurs 2000 32 1008 1015 11095242

